# Supplementary material for: Improved Route to Linear Triblock Copolymers by Coupling with Glycidyl Ether-Activated Poly(ethylene oxide) Chains
Source: Polymers (Basel). 2023 Apr 29;15(9):2128. doi: 10.3390/polym15092128 (PMC10180747; doi:10.3390/polym15092128)
Supplement: Supplementary file 1 [file polymers-15-02128-s001.zip › polymers-2326866-supplementary.pdf]

polymers

MDPI

Supplementary Materials

Table of Contents

Utilization of the Blocking Effect 2
Additional NMR Spectra 3
Comparison of Characteristic Signals in ^{1}H-NMR Spectra 7
Comparison of Proton Numbers 8
Full GPC Traces 8
DSC of EmPEO$_{1.9}$ 11
Exemplary Photo of a PI$_x$PS$_y$AmPEO$_{1.9}$ Membrane 11
Additional 2D SAXS Patterns 12
Details Concerning Fits to Radially Averaged SAXS Data 13
Sample Preparation by Cryo-Ultramicrotomy 14
References 14

Utilization of the Blocking Effect

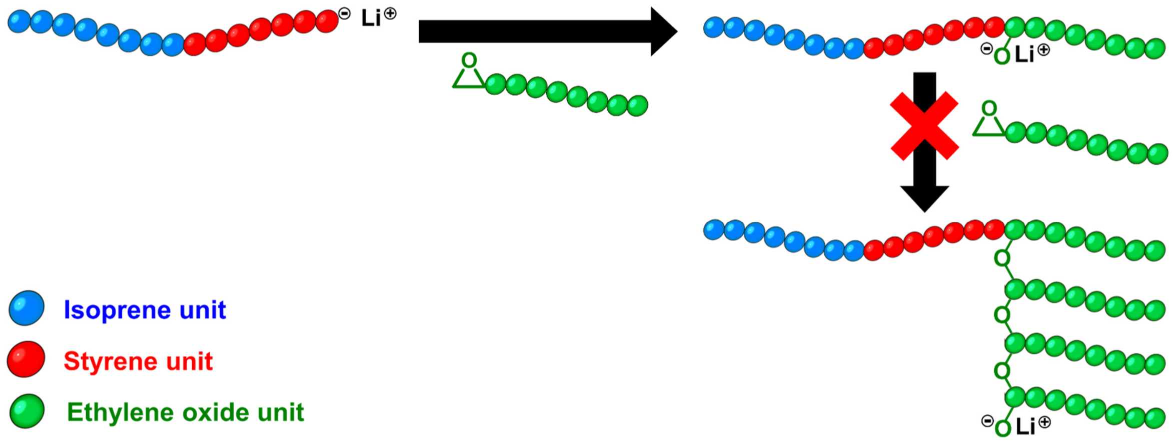

Scheme S1. Illustration of the utilization of the blocking effect for single EmPEO$_z$ chain attachment to the PI$_x$PS$_y^-$ anion with Li$^+$ as counterion due to the strong O-Li affinity.

Additional NMR Spectra

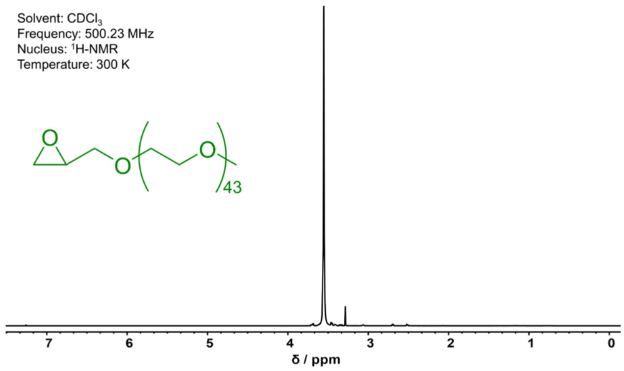

Figure S1. ^{1}H-NMR spectrum of $EmPEO_{1.9}$.

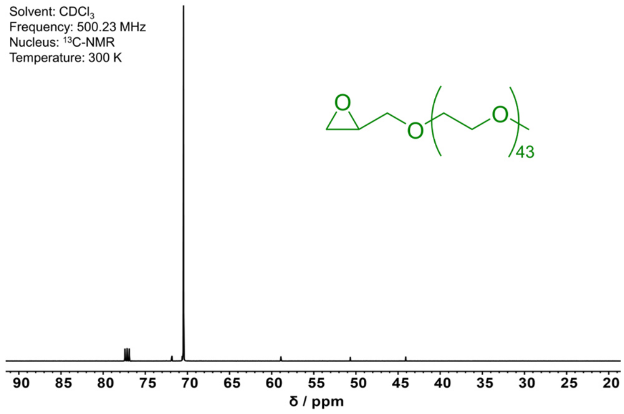

Figure S2. ^{13}C-NMR spectrum of $EmPEO_{1.9}$.

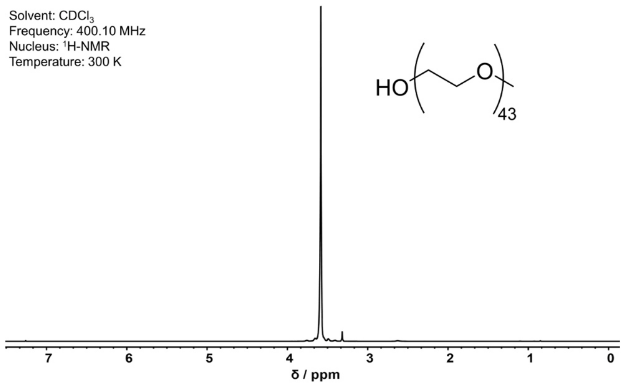

Figure S3. ^{1}H-NMR spectrum of $mPEO_{1.9}$.

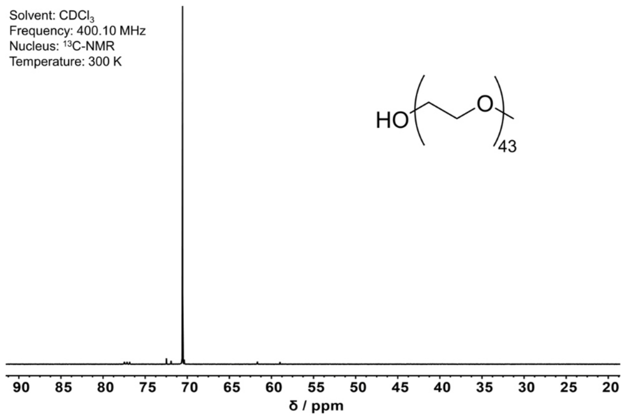

Figure S4. ^{13}C-NMR spectrum of $mPEO_{1.9}$.

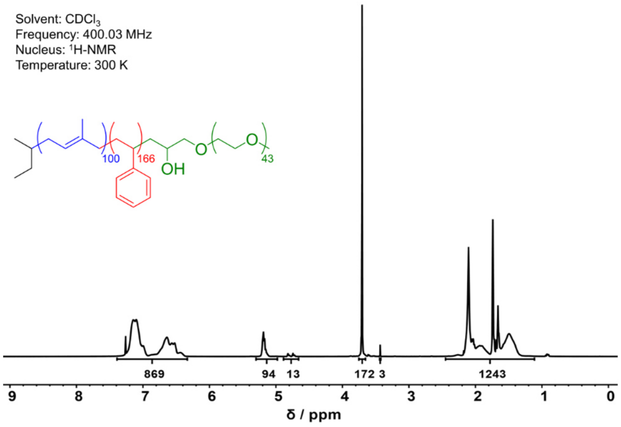

Figure S5. ^{1}H-NMR spectrum of $PI_{6.8}PS_{17.3}AmPEO_{1.9}$.

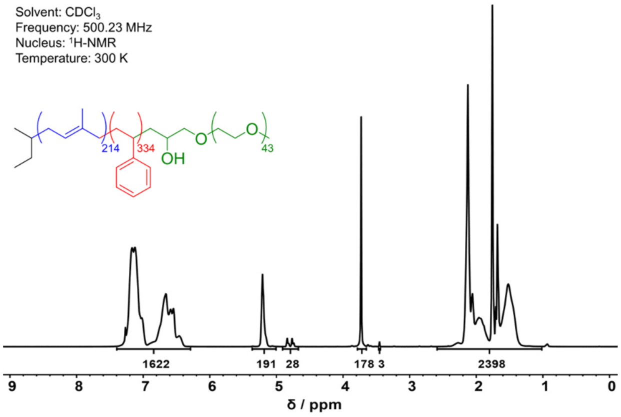

Figure S6. ^{1}H-NMR spectrum of $PI_{14.6}PS_{34.8}AmPEO_{1.9}$.

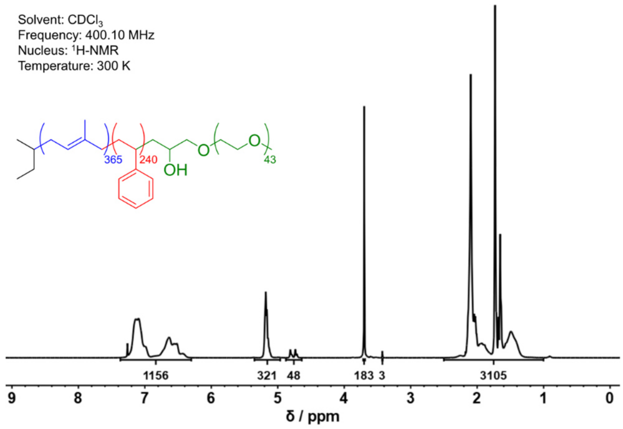

Figure S7. ^{1}H-NMR spectrum of $PI_{24.8}PS_{25.0}AmPEO_{1.9}$.

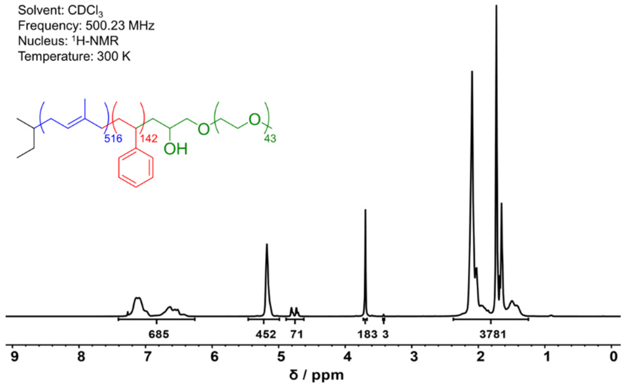

Figure S8. ^{1}H-NMR spectrum of $PI_{35.1}PS_{14.8}AmPEO_{1.9}$.

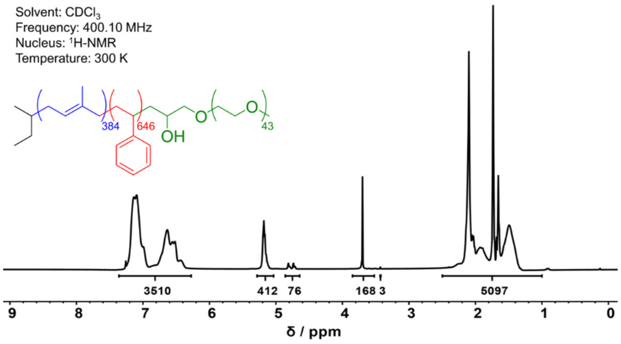

Figure S9. ^{1}H-NMR spectrum of $PI_{26.1}PS_{67.3}AmPEO_{1.9}$.

Comparison of Characteristic Signals in ^{1}H-NMR Spectra

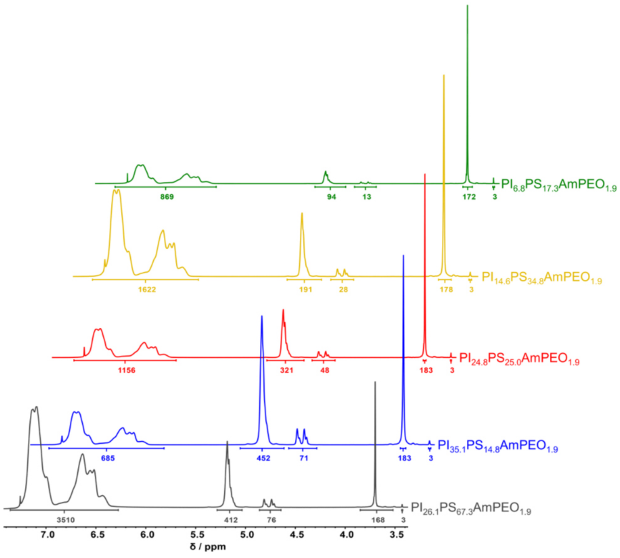

Figure S10. Comparison of the characteristic signals in the ^{1}H-NMR spectra of the synthesized $PI_xPS_yAmPEO_{1.9}$.

Comparison of Proton Numbers

Table S1. Comparison of the number of protons determined from ^{1}H-NMR integrals of the individual polymer blocks of the synthesized $PI_xPS_yAmPEO_{1.9}$ (cf. **Figure S10.**).

Polymer	$^1H_{1,4\text{-}PI_x}$ integral	$^1H_{3,4\text{-}PI_x}$ integral	$^1H_{PS_y}$ integral	$^1H_{PEO_{1.9}}$ integral
**$PI_{6.8}PS_{17.3}AmPEO_{1.9}$**	94	13	869	172
**$PI_{14.6}PS_{34.8}AmPEO_{1.9}$**	191	28	1622	178
**$PI_{24.8}PS_{25.0}AmPEO_{1.9}$**	321	48	1156	183
**$PI_{35.1}PS_{14.8}AmPEO_{1.9}$**	452	71	685	183
**$PI_{26.1}PS_{67.3}AmPEO_{1.9}$**	412	76	3510	168

Full GPC Traces

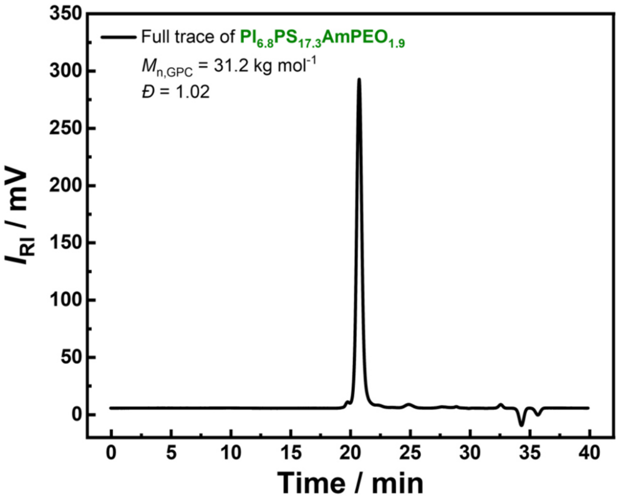

Figure S11. Full GPC trace of $PI_{6.8}PS_{17.3}AmPEO_{1.9}$.

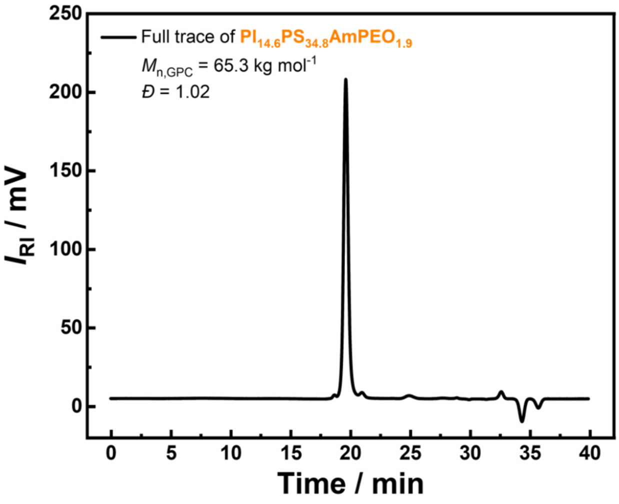

Figure S12. Full GPC trace of $PI_{14.6}PS_{34.8}AmPEO_{1.9}$.

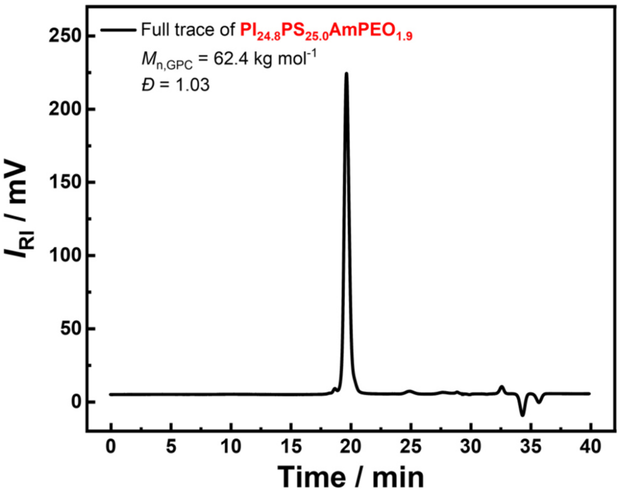

Figure S13. Full GPC trace of $PI_{24.8}PS_{25.0}AmPEO_{1.9}$.

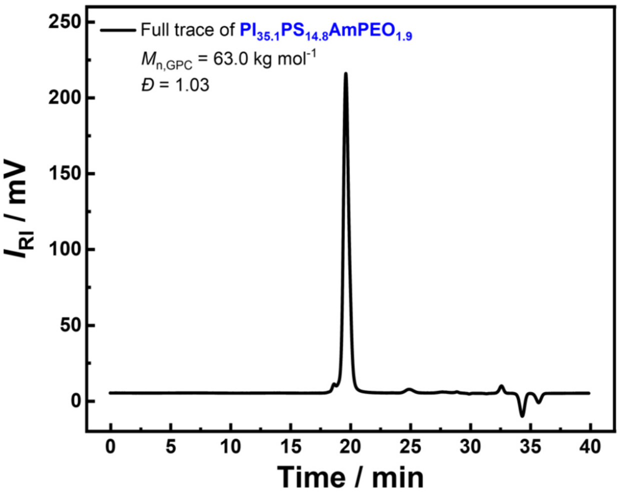

Figure S14. Full GPC trace of $PI_{35.1}PS_{14.8}AmPEO_{1.9}$.

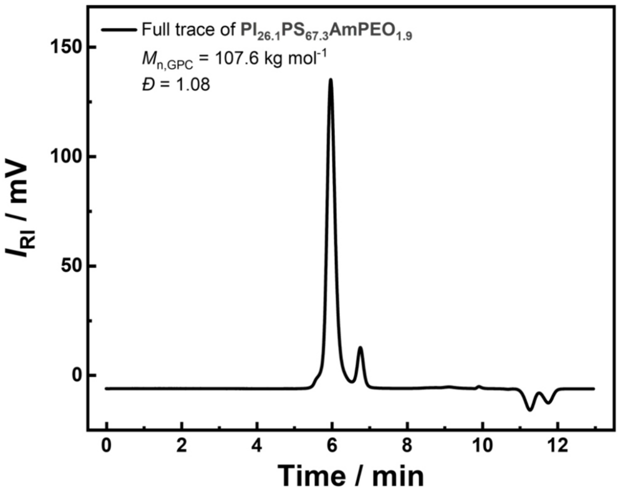

Figure S15. Full GPC trace of $PI_{26.1}PS_{67.3}AmPEO_{1.9}$.

DSC of $EmPEO_{1.9}$

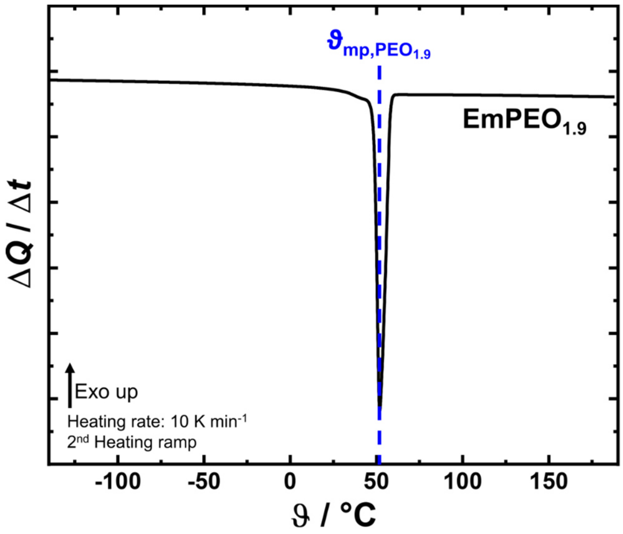

Figure S16. DSC measurement of $EmPEO_{1.9}$.

Exemplary Photo of a $PI_xPS_yAmPEO_{1.9}$ Membrane

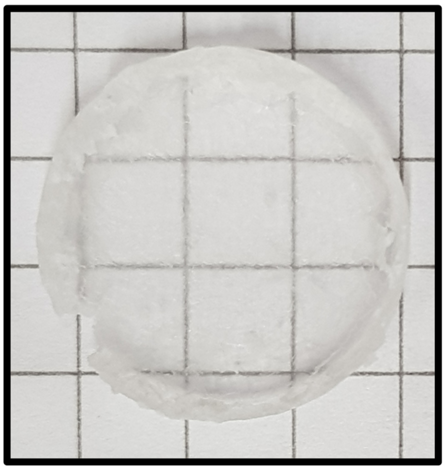

Figure S17. Exemplary photo of a colorless and transparent $PI_xPS_yAmPEO_{1.9}$ membrane cast from THF.

Additional 2D SAXS Patterns

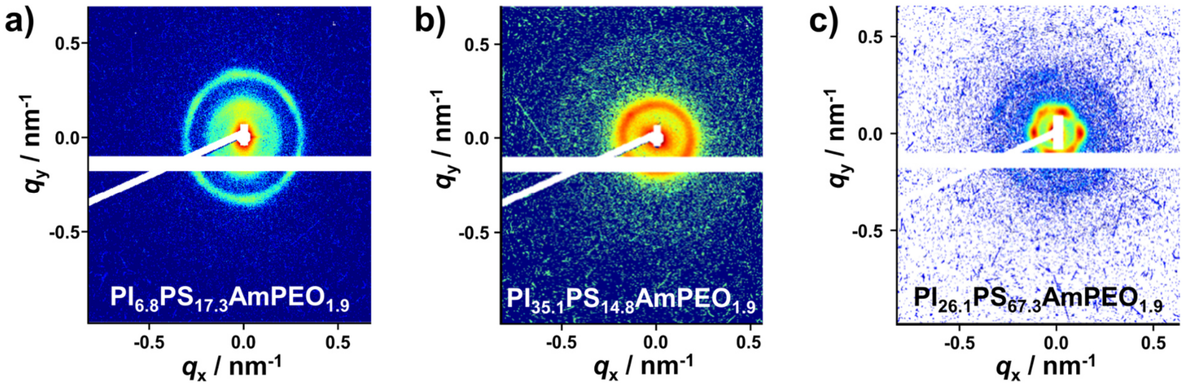

Figure S18. 2D SAXS patterns obtained at room temperature from the membrane in as-cast condition with a sample diameter of 2 mm of the synthesized $PI_{6.8}PS_{17.3}AmPEO_{1.9}$, $PI_{35.1}PS_{14.8}AmPEO_{1.9}$ and $PI_{26.1}PS_{67.3}AmPEO_{1.9}$.

Details Concerning Fits to Radially Averaged SAXS Data

The radially averaged data was fit with the program scatter [1,2]. Here the decoupling approximation is used which means the scattering curve is approximated as the sum of form and structure factor. This approach is valid if the particle size distribution is narrow and the spatial distribution is independent of the size distribution. Furthermore, for cylinders and lamellae the longitudinal contribution (length parallel to cylinder axis or normal to the lamella) can be decoupled as well, if it is much greater than the cross-sectional contribution, which in general is the case for mesoscopically ordered materials.

For a system of polydisperse particles ($b1$) distributed in a second phase ($b2$) with sharp interfaces the scattering per unit volume is given by:

$$I(q) = (b_1 - b_2)\rho_{\mathrm{N}}(\langle F^2(q)\rangle + \langle F(q)\rangle^2[\langle Z(q)\rangle - 1])$$

With $F(q)$ being the scattering amplitude of the particle form, ρ_{N} the number density of particles and with $Z(q)$ as the lattice factor which describes the spatial distribution of the particles. The angular brackets signify an average for either the size distribution with $F(q)$ or the spatial distribution with $Z(q)$. The lattice factor contains the symmetry related extinction rules for a given lattice as well as a factor for lattice disorder and the peak shape. For more details see [1] and [2].

Sample Preparation by Cryo-Ultramicrotomy

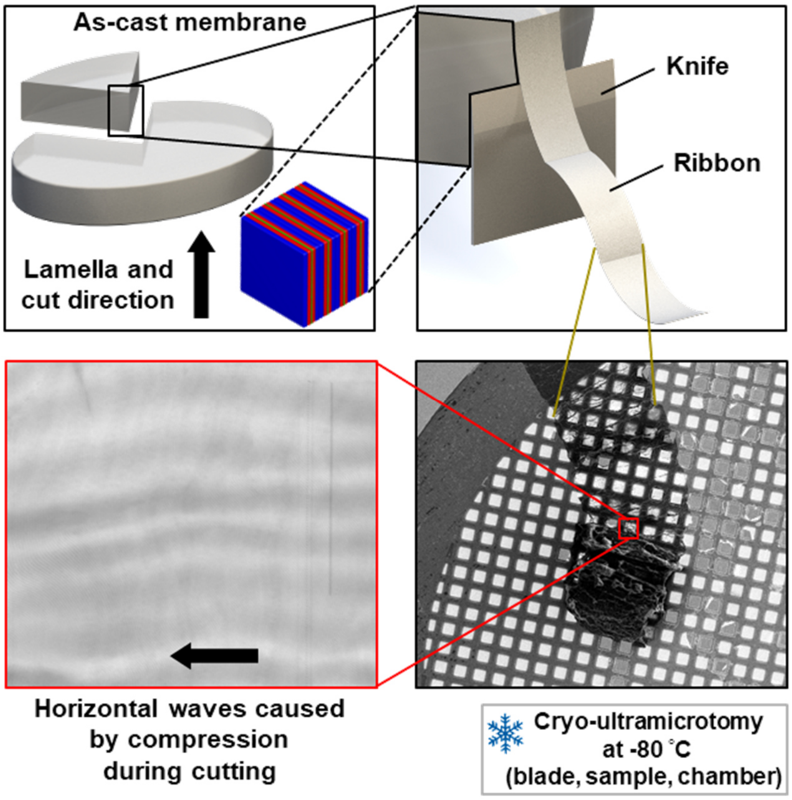

Scheme S2. Procedure of sample preparation by cryo-ultramicrotomy as well as sample placement onto the grid.

References

1. Förster, S.; Timmann, A.; Konrad, M.; Schellbach, C.; Meyer, A.; Funari, S.S.; Mulvaney, P.; Knott, R. Scattering Curves of Ordered Mesoscopic Materials. *The Journal of Physical Chemistry B* **2005**, *109*, 1347–1360.
2. Förster, S.; Fischer, S.; Zielske, K.; Schellbach, C.; Sztucki, M.; Lindner, P.; Perlich, J. Calculation of scattering-patterns of ordered nano- and mesoscale materials. *Advances in Colloid and Interface Science* **2011**, *163*, 53–83.
